# Tea Consumption and Risk of Bladder Cancer: A Dose-Response Meta-Analysis

**DOI:** 10.3389/fphys.2016.00693

**Published:** 2017-01-23

**Authors:** Hong Weng, Xian-Tao Zeng, Sheng Li, Joey S. W. Kwong, Tong-Zu Liu, Xing-Huan Wang

**Affiliations:** ^1^Center for Evidence-Based and Translational Medicine, Zhongnan Hospital of Wuhan UniversityWuhan, China; ^2^Department of Urology, Zhongnan Hospital of Wuhan UniversityWuhan, China; ^3^Chinese Evidence-Based Medicine Center and Chinese Cochrane Center, West China Hospital, Sichuan UniversityChengdu, China

**Keywords:** bladder cancer, dose-response, risk factor, tea consumption, meta-analysis

## Abstract

**Background and Objective:** Controversial results of the association between tea (black tea, green tea, mate, and oolong tea) consumption and risk of bladder cancer were reported among epidemiological studies. Thus, we performed a meta-analysis of observational studies to investigate the association.

**Methods:** We searched the PubMed and Embase for studies of tea consumption and bladder cancer that were published in any language up to March, 2016. Cohort or case-control studies were included in the meta-analysis. All statistical analyses were performed in Stata 12.0 software. Odds ratios (ORs) and 95% confidence intervals (CIs) were calculated to assess the relationship between tea consumption and risk of bladder cancer.

**Results:** Totally, 25 case-control studies (15 643 cases and 30 795 controls) and seven prospective cohort studies (1807 cases and 443 076 participants) were included. The meta-analysis showed that tea consumption was not significantly associated with bladder cancer risk (OR = 0.96, 95% CI 0.86–1.06) (in a comparison of highest vs. lowest category). No non-linearity association was observed between tea consumption and bladder cancer risk (*P* = 0.51 for non-linearity). Specific analysis for black tea, green tea, and mate yielded similar results. The dose-response analysis showed the summary OR for an increment of 1 cup/day of tea consumption was 1.01 (95% CI 0.97–1.05).

**Conclusion:** Results based on current meta-analysis indicated that no significant association was observed between tea consumption and risk of bladder cancer.

## Introduction

Bladder cancer is a very common disease worldwide, accounting for ~429 800 new cases and 165 100 deaths occurred in 2012 worldwide (Torre et al., 2015). Although bladder cancer incidence rates have been declining in most Western countries over the past decades, it remains an important and deadly cancer in the United States (Siegel et al., 2015). Bladder cancer incidence rates have been stable or declining over the past decades, and it may be owing to reductions in smoking prevalence, increasing the intake of fruits and vegetables, and schistosomiasis control and treatment (Chavan et al., 2014; Xu et al., 2015). However, feasible measures for the prevention of bladder cancer are still a miss. Therefore, more risk factors of bladder cancer should be identified for the prevention of bladder cancer.

Tea is a commonly consumed beverage worldwide. Previous research *in vitro* and *in vivo* has indicated that tea polyphenols present protective effects against some cancers including bladder cancer (Conde et al., 2014). Although the relationship between tea consumption and risk of bladder cancer is biologically plausible (Lu et al., 1999; Sagara et al., 2010), epidemiological studies on this theme have obtained inconsistent results. Two meta-analyses of observation studies concluded that tea consumption was not associated with an elevated risk of bladder cancer (Zeegers et al., 2001b; Qin et al., 2012). Nevertheless, a recently published meta-analysis suggested that tea consumption was associated with decreased risk of bladder cancer in Western countries (Zhang et al., 2015). In addition, one meta-analysis indicated that green tea reduced bladder cancer risk in Asians (Wang X. et al., 2013), and the other one suggested that high level of tea consumption in smokers was related to an elevated risk of bladder cancer and high level of black tea intake in females was related to a reduced risk of bladder cancer (Wu et al., 2013). Therefore, the relationship between tea intake and the risk of bladder cancer remains controversy. Additionally, none of above published meta-analyses performed a dose-response analysis. In order to clarify the relationship between tea consumption and the risk of bladder cancer, we performed the present dose-response meta-analysis of all published observational studies.

## Methods

### Eligible criteria

This study was conducted and reported following the preferred reporting items for systematic reviews and meta-analyses (PRISMA) statement (Moher et al., 2009). The inclusion criteria were as following: (1) case-control study or cohort study; (2) exposure was tea (including green tea, black tea, mate, and oolong tea) consumption; (3) outcome was incidence of bladder cancer; (4) study provided the odds ratios (ORs) or relative risks (RRs) with corresponding 95% confidence intervals (CIs) or data necessary to calculate them. When multiple papers reported on the same study were identified, the most informative or complete article would be included.

### Search strategy

PubMed and Embase were searched for studies examining the relationship between tea consumption and bladder cancer that were published in any language up to March, 2016. Search items including “tea,” “drink,” “beverage,” or “fluid” combined with “bladder cancer,” “bladder neoplasm,” “bladder tumor,” “bladder carcinoma,” “urothelium carcinoma,” or “transitional cell carcinoma” had been allied in the database retrieve. We also scanned the reference lists from all retrieved papers to identify additional studies. No restriction was applied.

### Data extraction

All data were extracted independently and crosschecked by two reviewers according to the pre-specified inclusion criteria. Discrepancy was resolved by discussion. The following information were extracted: First author, publication year, country, study period, sex, study design, type of control subjects for case-control studies, sample size, type of tea, consumption categories, the OR or RR with 95% CI for each category (the results adjusted with most potential confounders), and adjusted variables. Crude ORs or RRs with 95% CIs were only extracted when no adjusted ORs or RRs were presented. In addition, ORs or RRs with 95% CIs in different smoking status were also extracted for assessing the effect of smoking, which is an important confounding factor for bladder cancer.

### Methodological quality assessment

We performed methodological quality assessment of the included studies using the Newcastle-Ottawa Scale (Stang, 2010; Zeng X. et al., 2015), which is a nine-star scale contained three main items: Selection (0–4 stars), comparability (0–2 stars), and exposure (for cohort study, 0–3 stars), or outcome (for case-control study, 0–3 stars).

### Statistical analysis

The statistical analysis for the overall relationship between tea consumption and bladder cancer risk were based on random-effects model and on comparisons of the highest vs. lowest category of tea consumption (Zeng X. T. et al., 2015). The measure of interest is the OR with corresponding 95% CI. For studies reported the information by subsets (sex, smoking status, type of tea), we summarized the ORs with 95% CIs of the subsets in a fixed-effect model before aggregating them into overall analysis.

For dose-response analysis, we used the G-L method (Greenland, 1987; Orsini et al., 2012) to explore the relationship between tea consumption and bladder cancer risk. The potential non-linearity association was examined by modeling tea intake using restricted cubic splines with three knots at 10, 50, and 95% of the distribution. We assigned the median or middle point of the upper and lower boundaries in each category as the corresponding dose to the related OR for each study. If the highest category is open-ended, we assumed the both boundaries to be the same as the closest category. The lowest boundary was assumed to be zero if it was not present. Studies only reported three levels or more were included in the dose-response analysis.

The Cochran *Q* and *I*^2^ statistics were applied to detect statistical heterogeneity among studies (Higgins and Thompson, 2002). Heterogeneity was confirmed with a *P*-value of less than 0.1 or *I*^2^ value of more than 50%. To explore the potential heterogeneity among studies, stratified analyses were performed according to study design, sex, study location, smoking status, adjustment for age, and adjustment for smoking. In addition, subgroup analysis of type of tea had been carried out to further investigate the association between different type of tea consumption and the risk of bladder cancer. The meta-regression analysis was conducted to detect the between-group heterogeneity based on the aforementioned variables. Sensitivity analysis was performed by removing the studies that only provided crude ORs to examine the influence of these studies on the summarized Ors (Leng et al., 2015). Publication bias was detected by funnel plot and Egger's regression method (Egger et al., 1997). All statistical analysis was performed with Stata 12.0 (StataCorp, College Station, TX). All statistical tests were two-sided, with *P* < 0.05 considered statistically significant.

## Results

### Study characteristics

**Figure 1:**
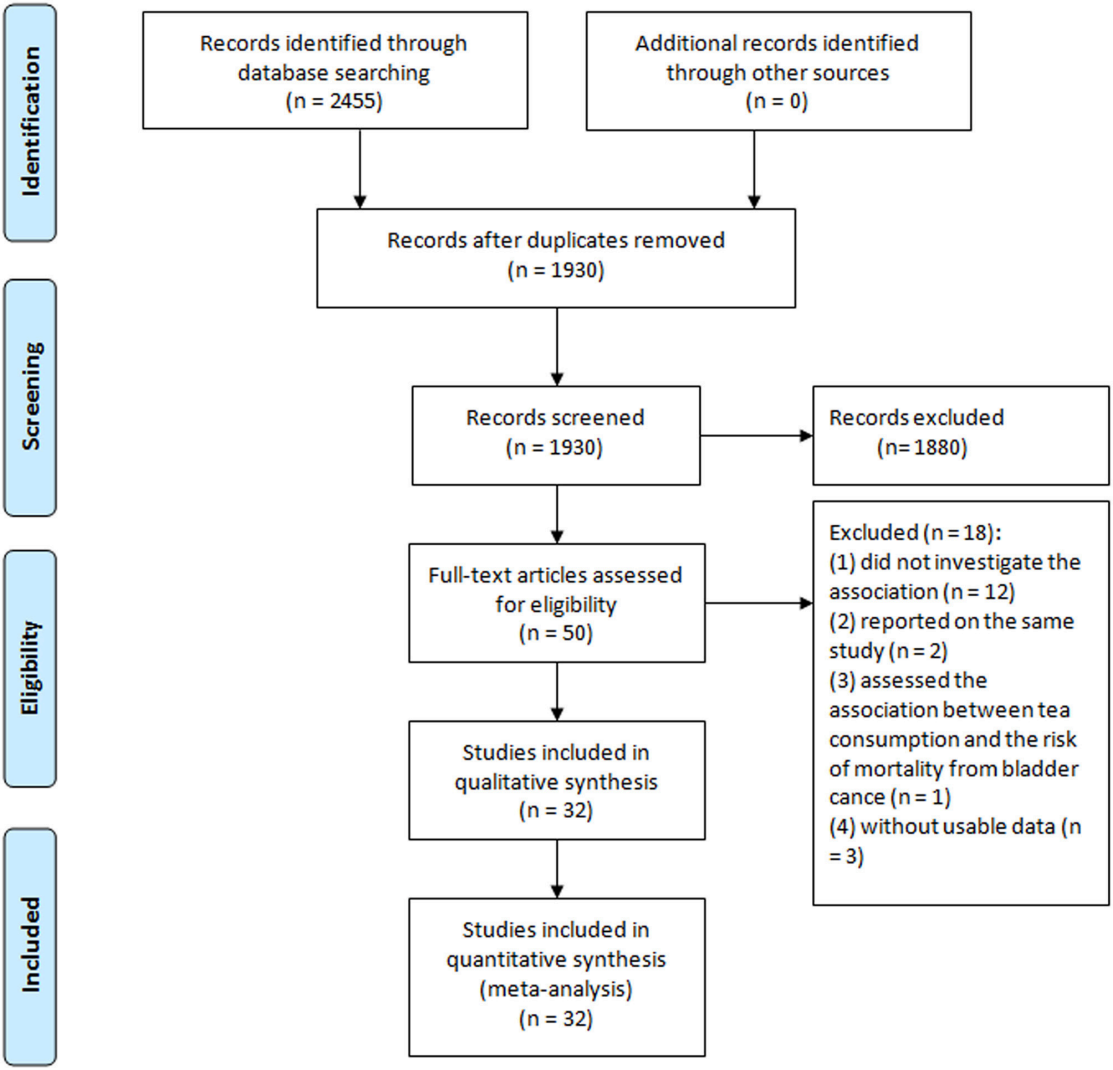
**Flow diagram of literature search and study selection**.

Detailed literature selection process was presented in Figure 1. A total of seven prospective cohort studies (Heilbrun et al., 1986; Chyou et al., 1993; Michaud et al., 1999; Nagano et al., 2000; Zeegers et al., 2001a; Kurahashi et al., 2009; Ros et al., 2011) included 443 076 participants, in which 1807 developed bladder cancer, and 25 case-control studies (Morgan and Jain, 1974; Howe et al., 1980; Hartge et al., 1983; Ohno et al., 1985; Jensen et al., 1986; Risch et al., 1988; Slattery et al., 1988; Clavel and Cordier, 1991; Nomura et al., 1991; D'Avanzo et al., 1992; Kunze et al., 1992; La Vecchia et al., 1992; Wilkens et al., 1996; Bruemmer et al., 1997; Lu et al., 1999; Bianchi et al., 2000; Geoffroy-Perez and Cordier, 2001; Woolcott et al., 2002; Wakai et al., 2004; Bates et al., 2007; De Stefani et al., 2007; Demirel et al., 2008; Jiang et al., 2008; Hemelt et al., 2010; Wang J. et al., 2013) including 15 643 cases and 30 795 controls, published from 1974 through 2013, were identified in the meta-analysis. Characteristics of included studies were presented in Table 1. Of the 32 studies, seven were cohort studies, 12 were hospital-based case-control studies, and 13 were population-based case-control studies. Of these studies, 11 conducted in US (Hartge et al., 1983; Heilbrun et al., 1986; Slattery et al., 1988; Nomura et al., 1991; Chyou et al., 1993; Wilkens et al., 1996; Bruemmer et al., 1997; Michaud et al., 1999; Bianchi et al., 2000; Jiang et al., 2008; Wang J. et al., 2013), 9 in Europe (Jensen et al., 1986; Clavel and Cordier, 1991; D'Avanzo et al., 1992; Kunze et al., 1992; La Vecchia et al., 1992; Geoffroy-Perez and Cordier, 2001; Zeegers et al., 2001a; Demirel et al., 2008; Ros et al., 2011), 4 in Canada (Morgan and Jain, 1974; Howe et al., 1980; Risch et al., 1988; Woolcott et al., 2002), 4 in Japan (Ohno et al., 1985; Nagano et al., 2000; Wakai et al., 2004; Kurahashi et al., 2009), two in China (Lu et al., 1999; Hemelt et al., 2010), one in Argentina (Bates et al., 2007) and one in Uruguay (De Stefani et al., 2007). The estimated quality of all included studies was in the rage of 4–8 stars.

**Table 1:** **Characteristics of studies of tea consumption and bladder cancer risk**.

**Study**	**Sex**	**Design**	**Study period**	**Country**	**Cases/controls, *n***	**Type of tea and consumption categories**	**OR/RR (95% CI)**	**Adjustments**	**NOS**
Morgan and Jain, 1974	Both	HCC	NR	Canada	232/232	Tea, cups/day (men)		None	4
						0	1.00		
						0.1–0.9	0.38 (0.10–1.43)		
						1.0–2.9	0.72 (0.24–2.14)		
						3.0–4.9	1.24 (0.35–4.41)		
						≥5	0.45 (0.13–1.62)		
						Tea, cups/day (women)			
						0	1.00		
						0.1–0.9	0.54 (0.25–1.17)		
						1.0–2.9	0.49 (0.26–0.94)		
						3.0–4.9	1.04 (0.51–2.13)		
						≥5	1.52 (0.65–3.53)		
Howe et al., 1980	Both	PCC	1974–1976	Canada	632/632	Tea (men)		None	5
						Never	1.0		
						Ever	1.0 (0.7–1.4)		
						Tea (women)			
						Never	1.0		
						Ever	0.5 (0.2–1.0)		
Hartge et al., 1983	Both	PCC	1977–1978	US	2982/5782	Tea, cups/wk (men)		Age, race, geographic area, tobacco, and coffee	6
						0	1.0		
						0.1–7	1.1 (0.8–1.4)		
						7.1–14	1.1 (0.7–1.5)		
						>14	1.0 (0.7–1.4)		
						Tea, cups/wk (women)			
						0	1.0		
						0.1–7	1.1 (0.7–1.7)		
						7.1–14	1.7 (1.0–2.9)		
						>14	1.2 (0.7–2.0)		
Ohno et al., 1985	Both	PCC	1976–1978	Japan	292/589	Black tea (men)		Age and smoking	7
						Not	1.00		
						Ever	0.95 (0.68–1.32)		
						Black tea (women)			
						Not	1.00		
						Ever	0.55 (0.29–1.03)		
Jensen et al., 1986	Both	PCC	1979–1981	Denmark	371/771	Tea, cups/day (men)		Smoking	6
						0	1.0		
						<2	0.8 (0.6–1.2)		
						2–4	2.1 (1.3–3.4)		
						4–6	1.5 (0.7–3.2)		
						Tea, cups/day (women)			
						0	1.0		
						<2	0.8 (0.4–1.5)		
						2–4	1.4 (0.6–3.0)		
						4–6	1.0 (0.4–2.5)		
Heilbrun et al., 1986	M	Cohort	1965–1985	US (Japanese ancestry)	57/7833	Black tea (men)		Age and smoking	8
						Almost never	1.0		
						<twice/wk	1.4 (0.77–2.54)		
						2–4 times/wk	1.0 (0.38–2.60)		
						>once/day	0.8 (0.33–1.94)		
Risch et al., 1988	Both	PCC	1979–1982	Canada	876/1668	Tea (men)		Life time cigarette consumption and history of diabetes	8
						Not	1.00		
						Average daily frequency (3/day)	1.04 (0.90–1.20)		
						Tea (women)			
						Not	1.00		
						Average daily frequency (3/day)	0.98 (0.78–1.22)		
Slattery et al., 1988	Both	PCC	1977–1982	US	419/889	Tea, 8-ounce servings/wk (Never smoked)		Age, sex, diabetes and bladder infections	8
						0 tea	1.00		
						1–3 cups tea	1.91 (0.99–3.68)		
						≥4 cups tea	2.25 (1.29–3.91)		
						Tea, 8-ounce servings/wk (Ever smoked)			
						0 tea	1.00		
						1–3 cups tea	0.82 (0.54–1.25)		
						≥4 cups tea	0.84 (0.55–1.29)		
Clavel and Cordier, 1991	Both	HCC	1984–1987	France	690/690	Tea, cups/day (men, Non-smokers)		Age, hospital and residence	6
						0	1.00		
						1	2.73 (0.86–8.67)		
						>1	0.48 (0.05–4.60)		
						Tea, cups/day (men, current smokers)			
						0	1.00		
						1	3.81 (0.83–6.69)		
						>1	1.46 (0.28–7.62)		
						Tea, cups/day (women, Non-smokers)			
						0	1.00		
						1	0.85 (0.28–2.60)		
						>1	1.04 (0.31–3.59)		
						Tea, cups/day (women, current smokers)			
						0	1.00		
						>0	0.19 (0.04–0.84)		
Nomura et al., 1991	Both	PCC	1977–1986	US (Caucasian and Japanese)	261/522	Tea, cup-years (men)		Smoking	8
						Non-drinkers	1.0		
						Drinkers	0.8 (0.5–1.4)		
						1–30	0.9 (0.5–1.5)		
						≥31	0.7 (0.4–1.3)		
						Tea, cup-years (women)			
						Non-drinkers	1.0		
						Drinkers	0.6 (0.2–1.5)		
						1–30	0.6 (0.2–1.6)		
						≥31	0.5 (0.2–1.8)		
						Black tea, cup-years (men)			
						Non-drinkers	1.0		
						Drinkers	1.0 (0.7–1.5)		
						1–10	1.2 (0.8–1.8)		
						≥11	0.7 (0.4–1.2)		
						Black tea, cup-years (women)			
						Non-drinkers	1.0		
						Drinkers	0.6 (0.3–1.3)		
						1–10	0.6 (0.2–1.3)		
						≥11	0.7 (0.3–1.7)		
D'Avanzo et al., 1992	Both	HCC	1985–1990	Italy	555/855	Tea		Age, sex, education, smoking, alcohol, and exposure to occupation	7
						Non-drinkers	1.0		
						Drinkers	0.9 (0.6–1.2)		
Kunze et al., 1992	Both	HCC	1977–1985	Germany	620/675	Black tea, cups/day (men)		Smoking	6
						0	1.0		
						1–2	1.1 (0.8–1.4)		
						3–4	1.4 (0.8–2.2)		
						≥5	1.4 (0.7–3.1)		
						Black tea, cups/day (women)			
						0	1.0		
						1–2	0.7 (0.3–1.4)		
						3–4	0.7 (0.3–1.8)		
						≥5	0.7 (0.2–2.3)		
La Vecchia et al., 1992	Both	HCC	1983–1990	Italy	365/6147	Tea		Age, sex, area of residence, education, smoking, and coffee	6
						Non-users	1.0		
						Users (≥1 cup/day)	0.8 (0.5–1.1)		
Chyou et al., 1993	Both	Cohort	1965–1985	US (Japanese ancestry)	96/7995	Green tea		Age and smoking	8
						Almost never	1.00		
						Ever	1.34 (0.79–2.27)		
						Black tea			
						Almost never	1.00		
						Ever	1.32 (0.87–2.00)		
Wilkens et al., 1996	Both	PCC	1979–1986	US (Caucasian and Japanese)	271/522	Tea (men)		Age, smoking, occupation, consumption of dark green vegetables in men, and total vitamin C consumption in women	8
						Q1 (low)	1.0		
						Q2	0.8 (0.5–1.4)		
						Q3	1.0 (0.6–1.7)		
						Q4 (high)	0.7 (0.4–1.3)		
						Tea (women)			
						Q1 (low)	1.0		
						Q2	0.6 (0.2–1.4)		
						Q3	0.7 (0.3–1.8)		
						Q4 (high)	0.9 (0.4–2.2)		
						Green tea (men)			
						Q1 (low)	1.0		
						Q2	1.1 (0.6–1.9)		
						Q3 (high)	1.1 (0.6–2.3)		
						Green tea (women)			
						Q1 (low)	1.0		
						Q2	0.8 (0.3–2.1)		
						Q3 (high)	0.9 (0.3–2.6)		
Bruemmer et al., 1997	Both	PCC	1987–1990	US	262/405	Tea, cups per day, wk, or mo (men)		Age, country, and smoking	8
						≤1/mo	1.0		
						>1/mo–1/wk	0.6 (0.3–1.2)		
						>1/wk–7/wk	0.9 (0.5–1.6)		
						>7/wk	2.5 (1.2–5.3)		
						Tea, cups per day, wk, or mo (women)			
						≤1/mo	1.0		
						>1/mo–1/wk	0.3 (0.1–1.1)		
						>1/wk–7/wk	0.8 (0.3–1.8)		
						>7/wk	0.9 (0.4–2.1)		
Lu et al., 1999	Both	HCC	1996–1997	China (Taiwan)	40/160	Tea		Age, sex, date of admission, family history, ethnicity, and smoking	8
						Non-drinkers	1.00		
						≤1/day	4.30 (0.51–35.88)		
						>1/day	2.77 (1.11–6.92)		
						Oolong tea			
						Non-drinkers	1.00		
						Drinkers	3.00 (1.20–7.47)		
Michaud et al., 1999	Both	Cohort	1986–1996	US	252/47909	Tea (1 cup)		Geographic region, age, smoking, energy intake, and intake of fruits and vegetables	6
						<1/mo	1.0		
						1/mo–4/wk	0.98 (0.74–1.29)		
						5/wk–1/day	0.74 (0.49–1.11)		
						≥2/day	0.69 (0.40–1.19)		
Bianchi et al., 2000	Both	PCC	1986–1989	US	1452/2434	Tea, cups/day		Age, sex, education, smoking, family history, occupation, beverage, chlorinated surface water, vegetable, and coffee	7
						None	1.0		
						<1	0.9 (0.7–1.1)		
						1–2.6	1.1 (0.9–1.3)		
						>2.6	0.9 (0.7–1.1)		
Nagano et al., 2000	Both	Cohort	1979–1981	Japan	114/38540	Green tea		Age, gender, radiation dose, smoking, education, BMI, and calendar time	6
						0–1/day	1.00		
						2–4/day	1.07 (0.61–2.00)		
						≥5/day	1.07 (0.58–2.08)		
						Black tea			
						0/wk	1.00		
						1/wk	0.79 (0.45–1.33)		
						≥2/wk	0.81 (0.43–1.44)		
Geoffroy-Perez and Cordier, 2001	Both	HCC	1984–1987	France	765/765	Tea, ml/wk (men)		Age, center, residence, and smoking	6
						0	1.00		
						1–950	1.42 (0.90–2.22)		
						>950	1.17 (0.72–1.90)		
						Tea, ml/wk (women)			
						0	1.00		
						1–950	0.91 (0.39–2.13)		
						>950	1.08 (0.50–2.32)		
Zeegers et al., 2001a	Both	Cohort	1986–1992	Netherland	569/3123	Tea, cups/day		Age, sex, smoking, coffee	6
						0	1.00		
						<2	0.64 (0.45–0.89)		
						2−>3	0.71 (0.53–0.93)		
						3−<4	0.51 (0.37–0.72)		
						4−<5	0.46 (0.34–0.64)		
						≥5	0.53 (0.39–0.74)		
Woolcott et al., 2002	Both	PCC	1992–1994	Canada	927/2118	Tea, cups/day		Age, sex, education, smoking, energy, calcium, fiber, and beer	7
						<1	1.00		
						1–2	1.18 (0.97–1.43)		
						3–4	1.15 (0.89–1.49)		
						≥5	1.31 (0.92–1.87)		
Wakai et al., 2004	Both	HCC	1994–2000	Japan	124/744	Green tea, cups/day		Age, sex, year of first visit, and cigarettes	6
						<1	1.00		
						1–4	1.40 (0.74–2.62)		
						5–9	2.67 (1.44–4.94)		
						≥10	1.18 (0.49–2.84)		
						Black tea, cups/day			
						Almost never	1.00		
						Occasionally	0.96 (0.60–1.53)		
						≥1	0.16 (0.02–1.14)		
Bates et al., 2007	Both	PCC	1996–2000	Argentina	114/114	Mate con bombilla, L/day (ever-smoker)		Age, sex, residence, education, cigarettes, and an indicator variable for whether or not the other type of mate was consumed at that time	6
						≤0.09	1.00		
						>0.09–0.36	1.36 (0.36–5.08)		
						>0.36–0.9	1.41 (0.57–3.46)		
						>0.9	1.16 (0.46–2.93)		
						Mate cocido, L/day (ever-smoker)			
						0	1.00		
						>0–<0.25	3.60 (0.31–41.3)		
						≥0.25	1.30 (0.65–2.60)		
De Stefani et al., 2007	Both	HCC	1996–2000	Uruguay	255/501	Tea, cups/day		Sex, age, residence, urban/rural status, education, family history, BMI, occupation, smoking, coffee, soft, and milk	6
						Never drinkers	1.0		
						<1	2.1 (1.4–3.1)		
						≥1	4.1 (1.7–9.9)		
						Mate, L/day			
						Never drinkers	1.0		
						0.1–0.9	1.3 (0.6–2.7)		
						1.0–1.9	2.1 (1.2–3.9)		
						≥2.0	3.7 (1.9–7.1)		
Demirel et al., 2008	Both	HCC	2001–2006	Turkey	164/324	Black tea	0.74 (0.38–1.43)	None	5
Jiang et al., 2008	Both	PCC	1987–1999	US	1586/1586	Tea, cups/day		Education, carotenoids, number of years as a hairdresser/barber, and smoking	5
						0	1.00		
						<1	0.96 (0.74–1.26)		
						1–2	0.95 (0.76–1.20)		
						3–4	1.16 (0.80–1.69)		
						≥5	0.88 (0.54–1.45)		
Kurahashi et al., 2009	Both	Cohort	1990–2005	Japan	206/104440	Green tea, cups/day (men)		Age, area, smoking, alcohol, and coffee	8
						<1	1.0		
						1–2	1.18 (0.73–1.91)		
						3–4	0.71 (0.43–1.18)		
						≥5	0.90 (0.56–1.45)		
						Green tea, cups/day (women)			
						<3	1.0		
						3–4	1.22 (0.49–3.00)		
						≥5	2.29 (1.06–4.92)		
Hemelt et al., 2010	Both	HCC	2005–2008	China	381/371	Green tea, cups/day		Age, sex, smoking	7
						No	1.00		
						<daily	0.83 (0.54–1.27)		
						Daily	1.02 (0.71–1.48)		
						<4	1.23 (0.76–1.97)		
						≥4	0.83 (0.53–1.28)		
						Black tea, cups/day			
						No	1.00		
						<daily	0.82 (0.56–1.22)		
						Daily	0.86 (0.59–1.25)		
						<4	0.82 (0.49–1.37)		
						≥4	0.88 (0.57–1.38)		
Ros et al., 2011	Both	Cohort	1992–2000	European countries	513/233236	Tea, ml/day		Age, sex, center, smoking, and energy intake from fat and Non-fat sources	8
						<12 for men; <16 for women	1.00		
						12–199 for men; 16–263 for women	1.09 (0.78–1.52)		
						≥200 for men; ≥264 for women	0.91 (0.64–1.30)		
Wang J. et al., 2013	Both	HCC	1999–2007	US	1007/1299	Tea, cups/day		Age, sex, ethnicity, energy intake, and smoking	7
						Never	1.00		
						0.1–0.70	0.74 (0.59–0.92)		
						≥0.71	0.65 (0.53–0.81)		
						Black tea, cups/day			
						Never	1.00		
						0.1–0.56	0.71 (0.57–0.88)		
						≥0.57	0.67 (0.54–0.83)		
						Green tea, cups/day			
						Never	1.00		
						0.1–0.13	0.82 (0.61–1.11)		
						≥0.14	0.60 (0.45–0.79)		

*BMI, body mass index; HCC, hospital-based case-control; PCC, population-based case-control; NR, not report; OR, odds ratio; RR, relative risk; CI, confidence interval; NOS, Newcastle-Ottawa Scale*.

### Tea consumption and bladder cancer

**Figure 2:**
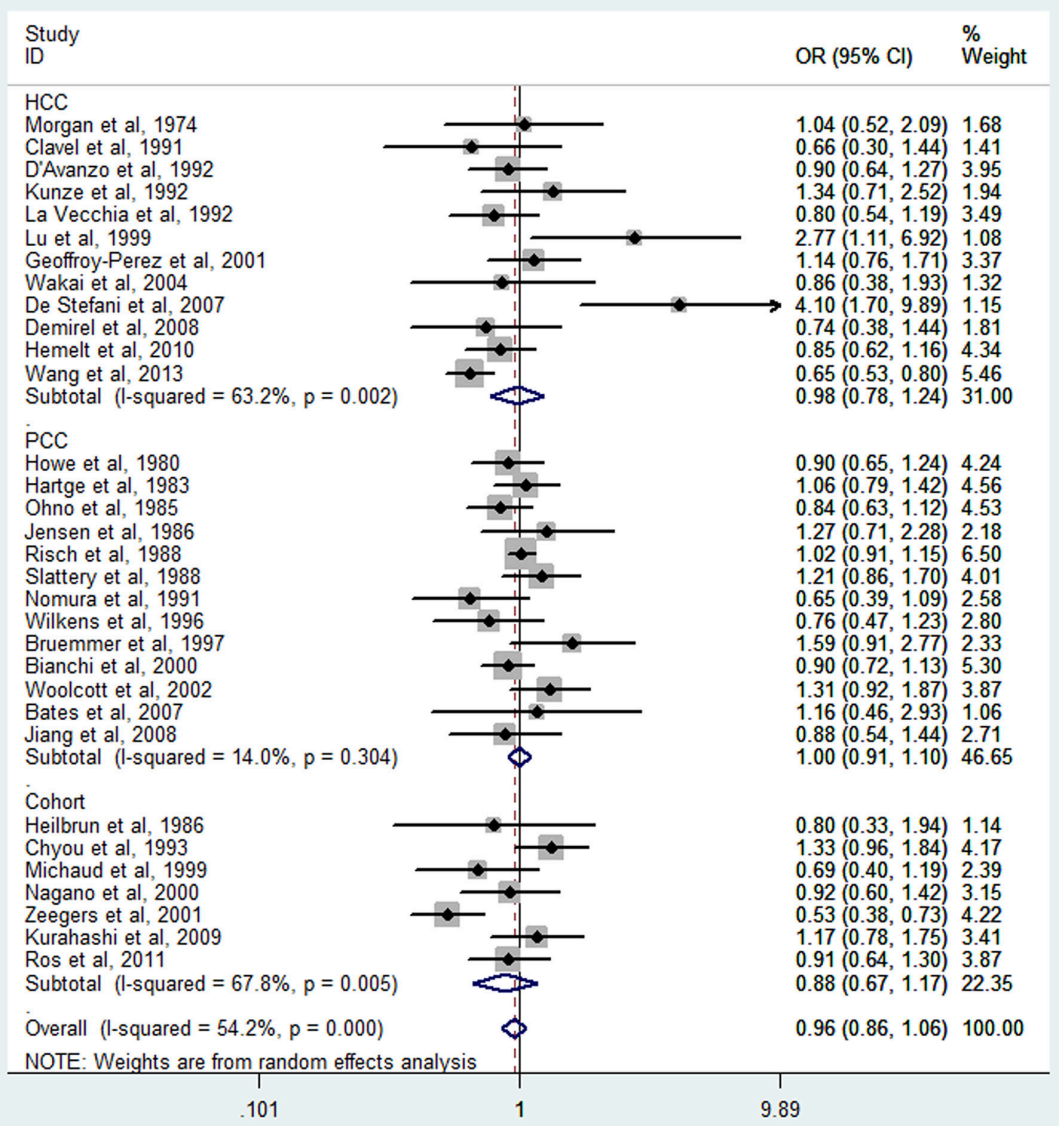
**The forest plot of tea consumption and the risk of bladder cancer**.

The meta-analysis of all 32 studies, no significant association was observed between high tea consumption and risk of bladder cancer (highest vs. lowest: OR = 0.96, 95% CI 0.86–1.06) (Figure 2), with moderate to high heterogeneity (*I*^2^ = 54.2%, *P*_heterogeneity_). For cohort studies, the pooled OR was 0.88 (95% CI 0.67–1.17), with certain between-study heterogeneity (*I*^2^ = 67.8%, *P*_heterogeneity_ = 0.005) (Table 2). For hospital-based case-control studies, the pooled OR was 0.98 (95% CI 0.78–1.24), with certain evidence of between-study heterogeneity (*I*^2^ = 63.2%, *P*_heterogeneity_). For population-based case-control studies, the pooled OR was 1.00 (95% CI 0.91–1.10), with no evidence of between-study heterogeneity (*I*^2^ = 14.0%, *P*_heterogeneity_).

**Table 2:** **Meta-analysis of tea consumption and bladder cancer risk**.

**Type of tea and subgroups**	**No. of studies**	**Test of association**		***I*****^2^ (%)**	***P*****-for heterogeneity**	
		**OR (95% CI)**	***P*****-value**		***P*****-for within group**	***P*****-for between group**
Tea	32	0.96 (0.86–1.06)	0.39	54.2	<0.001	–
**STUDY DESIGN**						
HCC	12	0.98 (0.78–1.24)	0.90	63.2	0.002	0.45
PCC	13	1.00 (0.91–1.10)	0.93	14.0	0.30	
Cohort	7	0.88 (0.67–1.17)	0.38	67.8	0.005	
**SEX**						
Male	14	1.02 (0.92–1.13)	0.75	1.3	0.44	0.51
Female	13	0.93 (0.75–1.16)	0.51	22.8	0.21	
**STUDY LOCATION**						
America	17	0.99 (0.86–1.15)	0.94	61.9	<0.001	0.83
Europe	9	0.87 (0.70–1.07)	0.17	46.8	0.06	
Asia	6	0.97 (0.78–1.21)	0.78	33.5	0.02	
**ADJUSTMENT FOR AGE**						
Yes	24	0.97 (0.84–1.10)	0.61	62.0	<0.001	0.75
No	8	0.99 (0.90–1.09)	0.86	0	0.57	
**ADJUSTMENT FOR SMOKING**						
Yes	27	0.96 (0.86–1.08)	0.49	59.5	<0.001	0.86
No	5	0.97 (0.79–1.19)	0.79	0	0.49	
**SMOKING STATUS**						
Non-smokers	2	1.54 (0.60–3.94)	0.37	60.9	0.11	0.26
Smokers	3	0.91 (0.61–1.36)	0.65	25.2	0.26	
Black tea	10	0.84 (0.70–1.01)	0.06	34.9	0.13	–
**STUDY DESIGN**						
HCC	5	0.79 (0.58–1.08)	0.14	42.8	0.14	0.28
PCC	2	0.80 (0.62–1.02)	0.07	0	0.51	
Cohort	3	1.06 (0.75–1.50)	0.73	9.3	0.33	
**STUDY LOCATION**						
America	4	0.83 (0.58–1.18)	0.30	63.5	0.04	0.42
Europe	2	1.00 (0.56–1.80)	0.99	38.0	0.20	
Asia	4	0.83 (0.66–1.04)	0.10	0	0.45	
**ADJUSTMENT FOR AGE**						
Yes	7	0.83 (0.66–1.05)	0.12	44.9	0.09	0.60
No	3	0.86 (0.58–1.27)	0.44	29.2	0.24	
**ADJUSTMENT FOR SMOKING**						
Yes	9	0.85 (0.69–1.04)	0.11	41.8	0.09	0.40
No	1	0.74 (0.38–1.44)	0.37	–	–	
Green tea	7	0.95 (0.73–1.24)	0.71	52.5	0.05	–
**STUDY DESIGN**						
HCC	3	0.73 (0.53–1.00)	0.052	34.5	0.22	0.51
PCC	1	1.04 (0.59–1.84)	0.89	–	–	
Cohort	3	1.20 (0.90–1.59)	0.22	0	0.86	
**STUDY LOCATION**						
America	3	0.90 (0.53–1.54)	0.71	75.9	0.02	0.79
Asia	4	1.03 (0.53–1.54)	0.85	0	0.70	
Mate	2	2.17 (0.70–6.74)	0.18	75.0	0.05	–
Oolong tea	1	3.00 (1.20–7.47)	0.02	–	–	–

*HCC, hospital-based case-control; PCC, population-based case-control; OR, odds ratio; CI, confidence interval*.

**Figure 3:**
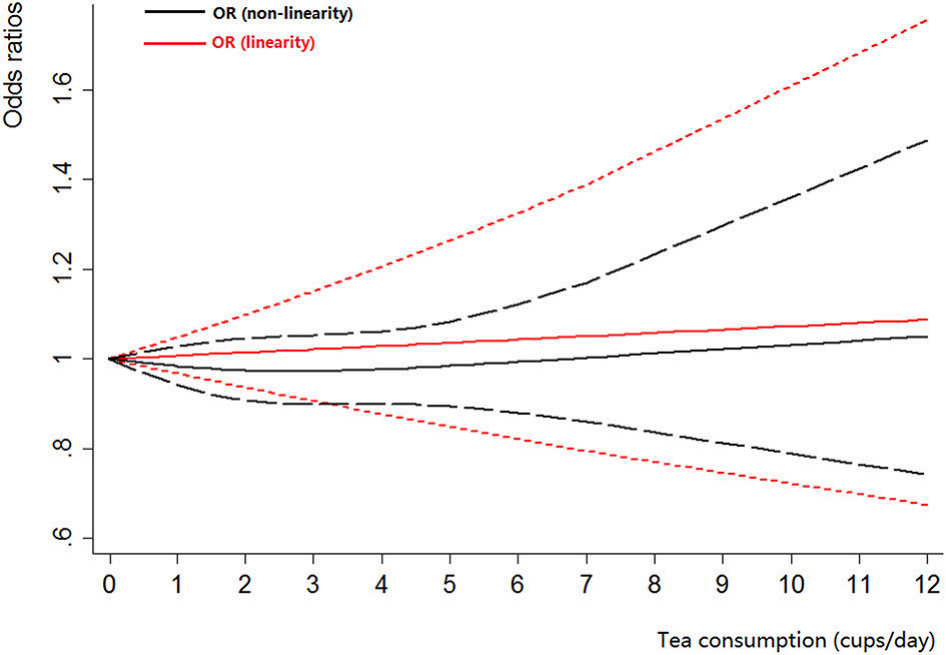
**The dose-response analysis of tea consumption and the risk of bladder cancer**. The black solid line and the black long dashed line represent the estimated RRs and corresponding 95% CIs for the non-linearity. The red solid line and the red short dashed line represent the estimated RRs and corresponding 95% CIs for the linearity.

Four cohort studies (Michaud et al., 1999; Nagano et al., 2000; Zeegers et al., 2001a; Kurahashi et al., 2009) and 12 case-control studies (Morgan and Jain, 1974; Hartge et al., 1983; Jensen et al., 1986; Clavel and Cordier, 1991; Kunze et al., 1992; Lu et al., 1999; Bianchi et al., 2000; Woolcott et al., 2002; Wakai et al., 2004; Jiang et al., 2008; Hemelt et al., 2010; Wang J. et al., 2013) were included for the dose-response meta-analysis of tea consumption. The results had been shown in Figure 3. There was no evidence of a non-linearity association between tea consumption and bladder cancer risk (*P*_non-linearity_ = 0.51). Thus, a linear regression model was applied. The summary OR of bladder cancer for an increase of one cup of tea per day was 1.01 (95% CI 0.97–1.05, *P*_linear_ = 0.73). For cohort studies, no non-linearity association was observed (*P*_non-linearity_ = 0.11). The summary OR of bladder cancer for an increase of 1 cup/day of tea was 0.97 (95% CI 0.88–1.06, *P*_linear_ = 0.47). For case-control studies, we found no evidence of a non-linearity association between tea consumption and bladder cancer risk (*P*_non-linearity_ = 0.60). We then used the linear model among case-control studies. The pooled OR of bladder cancer for an increase of 1 cup/day was 1.02 (95% CI 0.98–1.06, *P*_linear_ = 0.27).

### Black tea consumption and bladder cancer

**Figure 4:**
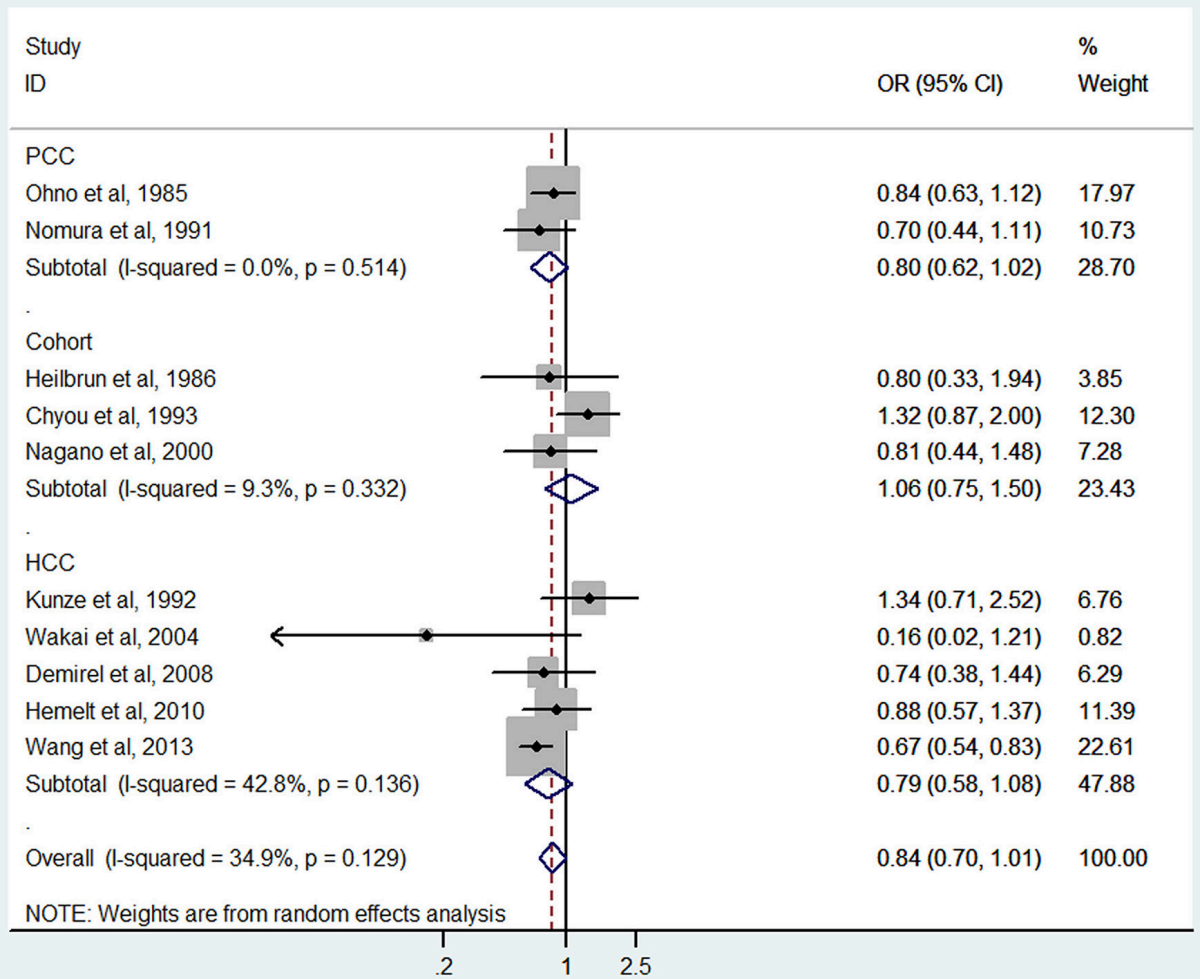
**The forest plot of black tea consumption and the risk of bladder cancer**.

Black tea consumption was examined in 3 cohort studies (Heilbrun et al., 1986; Chyou et al., 1993; Nagano et al., 2000), 5 hospital-based case-control studies (Kunze et al., 1992; Wakai et al., 2004; Demirel et al., 2008; Hemelt et al., 2010; Wang J. et al., 2013), and 2 population-based case-control studies (Ohno et al., 1985; Nomura et al., 1991). No significant association was observed between black tea consumption and risk of bladder cancer (OR = 0.84, 95% CI 0.70–1.01) (Figure 4), with moderate between-study heterogeneity (*I*^2^ = 34.9%, *P*_heterogeneity_ = 0.13). For cohort studies, the combined OR was 1.06 (95% CI 0.75–1.50), with low between-study heterogeneity (*I*^2^ = 9.3%, *P*_heterogeneity_ = 0.33) (Table 2). For hospital-based case-control studies, the pooled OR was 0.79 (95% CI 0.58–1.08), with low to moderate between-study heterogeneity (*I*^2^ = 42.8%, *P*_heterogeneity_ = 0.14). For population-based case-control studies, the combined OR was 0.80 (95% CI 0.62–1.02), with no between-study heterogeneity (*I*^2^ = 0.0%, *P*_heterogeneity_ = 0.51).

**Figure 5:**
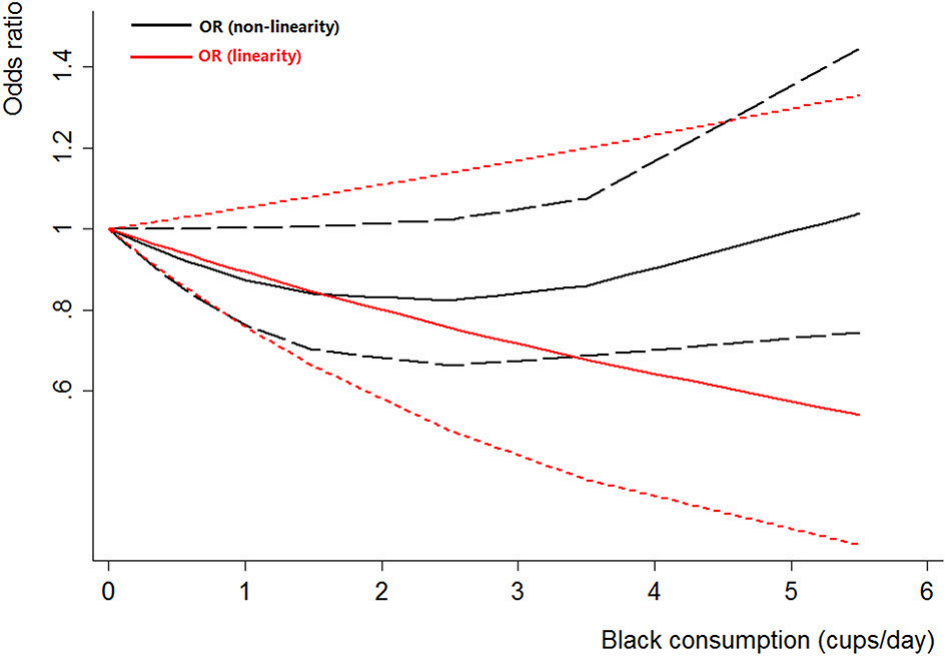
**The dose-response analysis of black tea consumption and the risk of bladder cancer**. The black solid line and the black long dashed line represent the estimated RRs and corresponding 95% CIs for the non-linearity. The red solid line and the red short dashed line represent the estimated RRs and corresponding 95% CIs for the linearity.

Four case-control studies (Kunze et al., 1992; Wakai et al., 2004; Hemelt et al., 2010; Wang J. et al., 2013) were included for the dose-response meta-analysis of black tea consumption. The results had been shown in Figure 5. There was no evidence of a non-linearity association between black tea consumption and bladder cancer risk (*P*_non-linearity_ = 0.06). A linear model was used among these studies. The poled OR of bladder cancer for an increase of 1 cup/day of black tea was 0.89 (95% CI 0.76–1.05, *P*_linear_ = 0.18).

### Green tea consumption and bladder cancer

**Figure 6:**
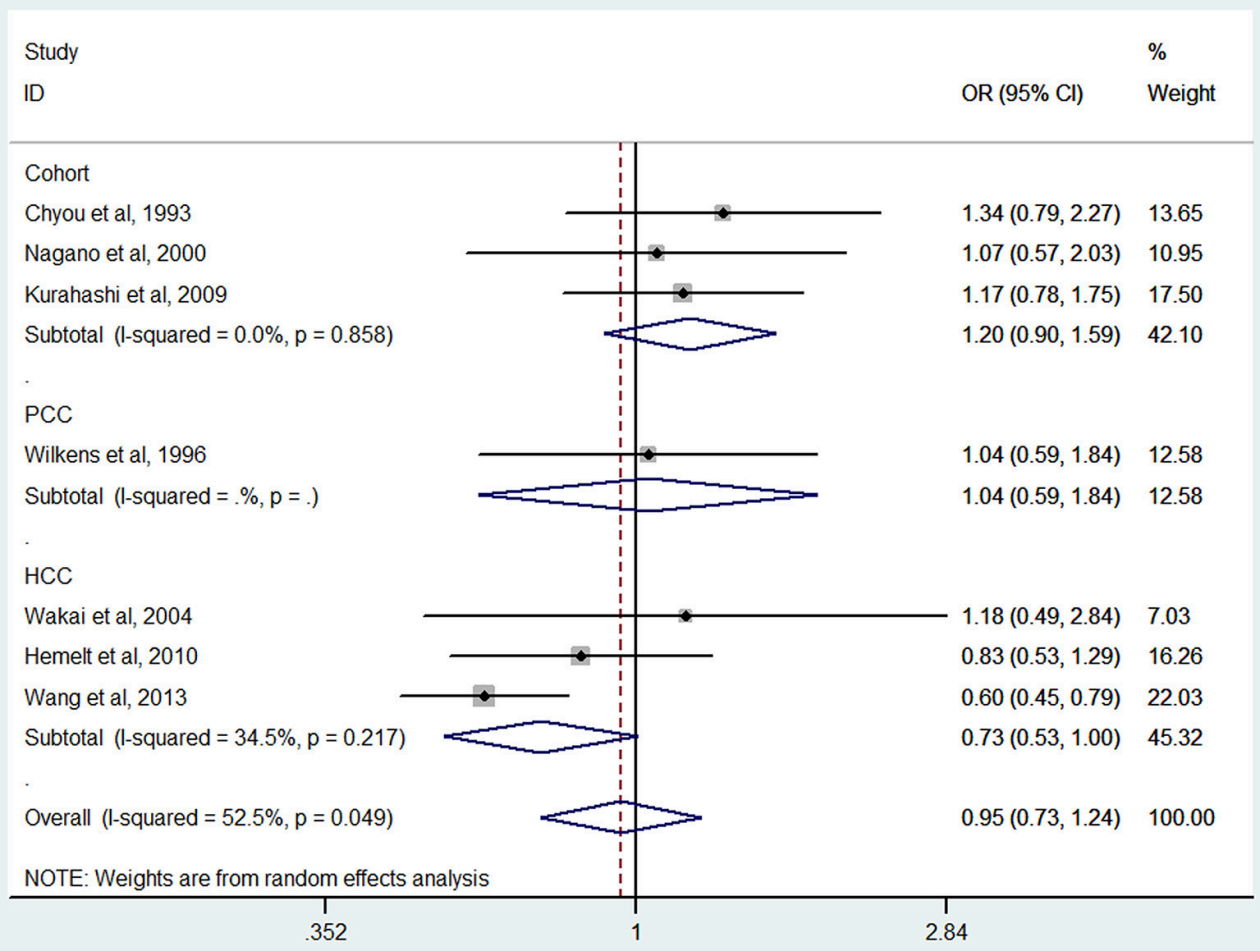
**The forest plot of green tea consumption and the risk of bladder cancer**.

Green tea consumption was assessed in 3 cohort studies (Chyou et al., 1993; Nagano et al., 2000; Kurahashi et al., 2009), 3 hospital-based case-control studies (Wakai et al., 2004; Hemelt et al., 2010; Wang J. et al., 2013), and one population-based case-control study (Wilkens et al., 1996). No significant association was observed between green tea consumption and bladder cancer risk (OR = 0.95, 95% CI 0.73–1.24) (Figure 6), with moderate heterogeneity (*I*^2^ = 52.5%, *P*_heterogeneity_ = 0.05). For cohort studies, the combined OR was 1.20 (95% CI 0.90–1.59), with no heterogeneity (*I*^2^ = 0.0%, *P*_heterogeneity_ = 0.86) (Table 2). For hospital-based case-control studies, the pooled OR was 0.73 (95% CI 0.53–1.00), with moderate between-study heterogeneity (*I*^2^ = 34.5%, *P*_heterogeneity_ = 0.22). For population-based case-control studies, the combined OR was 1.04 (95% CI 0.59–1.84).

**Figure 7:**
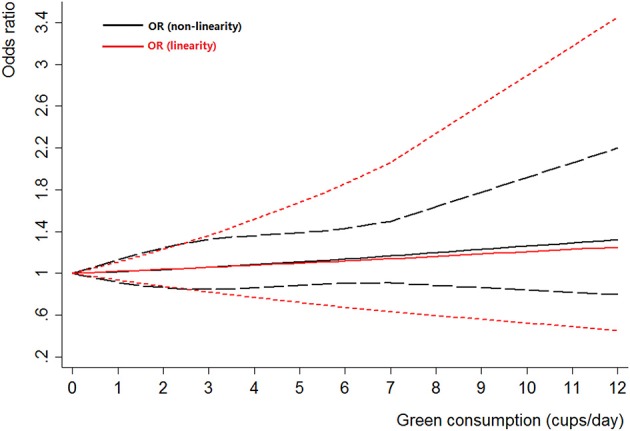
**The dose-response analysis of green tea consumption and the risk of bladder cancer**. The black solid line and the black long dashed line represent the estimated RRs and corresponding 95% CIs for the non-linearity. The red solid line and the red short dashed line represent the estimated RRs and corresponding 95% CIs for the linearity.

Two cohort studies (Nagano et al., 2000; Kurahashi et al., 2009) and three case-control studies (Wakai et al., 2004; Hemelt et al., 2010; Wang J. et al., 2013) were included for the dose-response meta-analysis of green tea consumption. The results had been shown in Figure 7. No evidence of non-linearity association was detected between green tea consumption and bladder cancer risk (*P*_non-linearity_ = 0.92). A linear model suggested that the summary OR of bladder cancer for an increase of 1 cup/day of green tea was 1.02 (95% CI 0.94–1.1, *P*_linear_ = 0.66).

### Mate and oolong tea consumption and risk of bladder cancer

Mate consumption was investigated in 2 case-control studies (Bates et al., 2007; De Stefani et al., 2007). No significant association was observed between mate consumption and bladder cancer risk (OR = 2.17, 95% CI 0.70–6.74), with moderate to high between-study heterogeneity (*I*^2^ = 75.0%, *P*_heterogeneity_ = 0.05) (Table 2).

Oolong tea consumption was examined in only one study (Lu et al., 1999). An elevated risk was observed between oolong tea consumption and bladder cancer risk (OR = 3.00, 95% CI 1.20–7.47) (Table 2).

### Subgroup and meta-regression analyses

**Figure 8:**
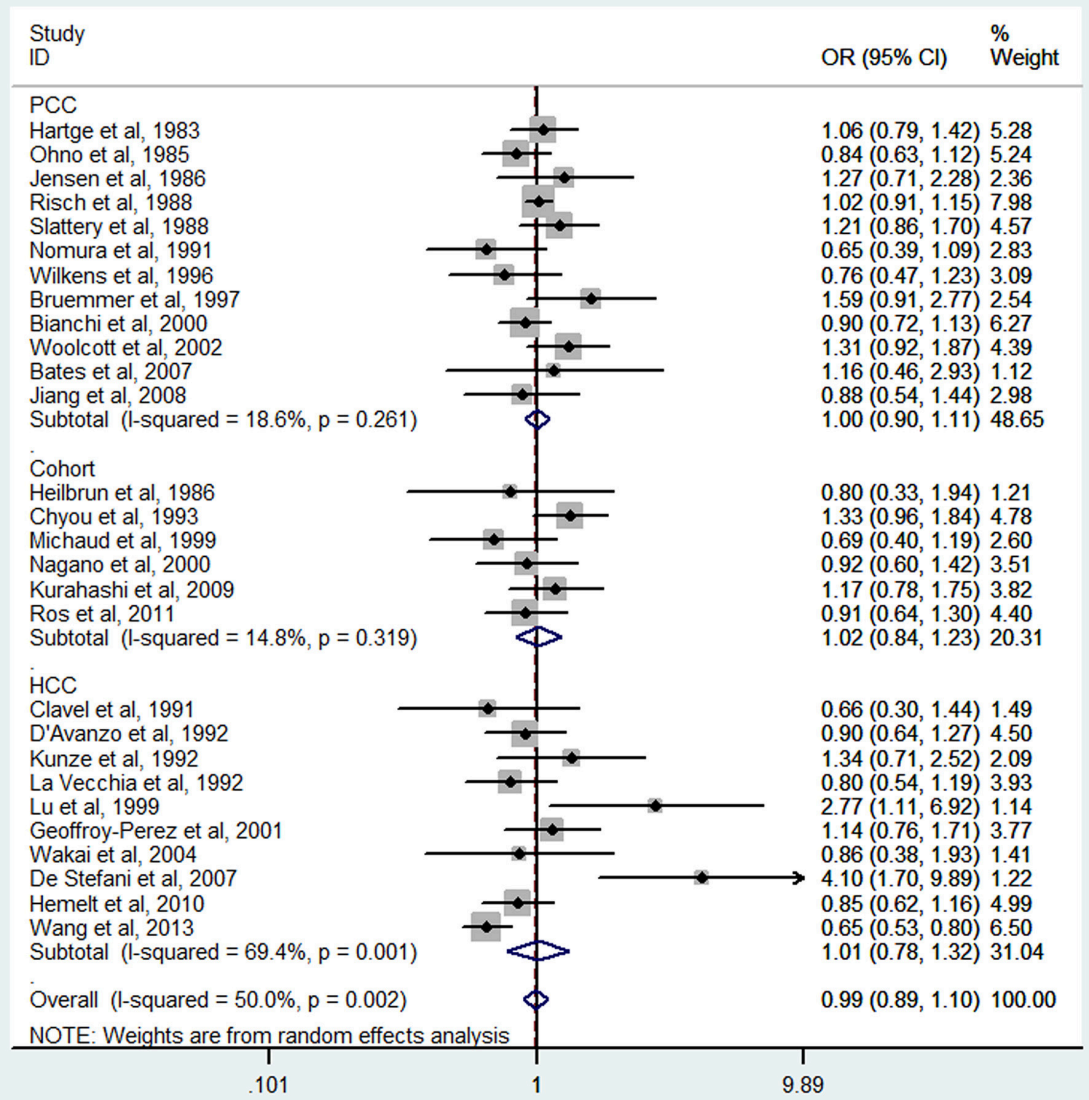
**Sensitivity analysis of tea consumption and risk of bladder cancer**.

Results of subgroup analyses had been shown in Table 2. For tea consumption and risk of bladder cancer, subgroup analyses defined by study design, sex, study location, adjustment for age, adjustment for smoking, and smoking status did not show any substantial change in the summary OR, with no evidence of between-group heterogeneity by meta-regression analysis (Table 2). For black tea consumption and risk of bladder cancer, subgroup analyses defined by study design, study location, adjustment for age, and adjustment for smoking did not show any substantial change in the summary OR, with no evidence of between-group heterogeneity by meta-regression analysis (Table 2). For green tea consumption and risk of bladder cancer, subgroup analyses defined by study design and study location did not show any substantial change in the summary OR, with no evidence of between-group heterogeneity by meta-regression analysis (Table 2). Sensitivity analysis by removing four studies (Morgan and Jain, 1974; Howe et al., 1980; Zeegers et al., 2001a; Demirel et al., 2008) with crude ORs did not change the overall results of tea consumption among all studies(OR = 0.99, 95% CI 0.89–1.10), cohort studies (OR = 1.02, 95% CI 0.84–1.23), hospital-based case-control studies (OR = 1.01, 95% CI 0.78–1.32), and population-based case-control studies (OR = 1.00, 95% CI 0.90–1.11),; but the heterogeneity was reduced in cohort studies (*I*^2^ = 14.8%, *P* = 0.32 for heterogeneity) (Figure 8).

### Publication bias

**Figure 9:**
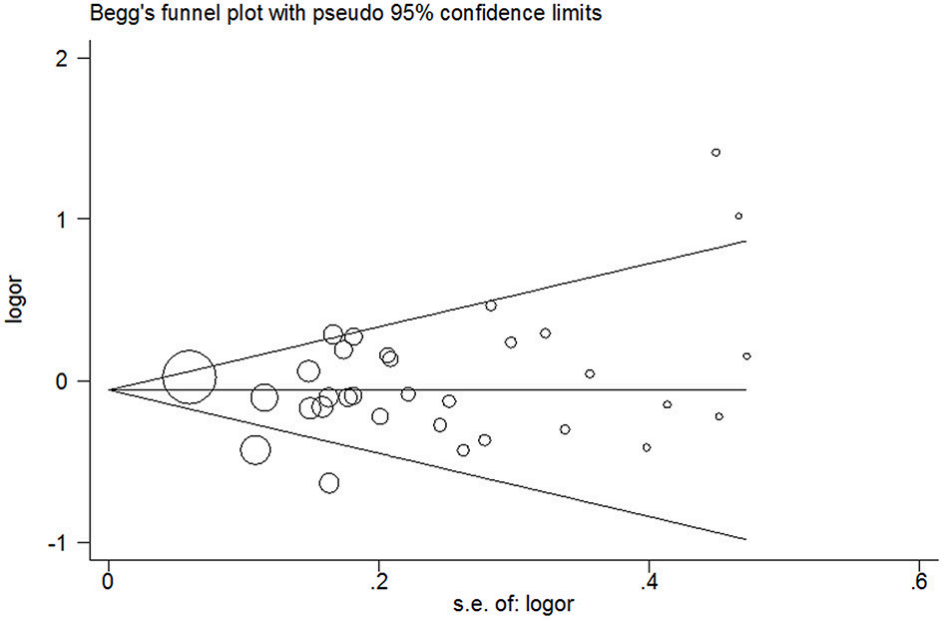
**Funnel plot for tea consumption and risk of bladder cancer**.

**Figure 10:**
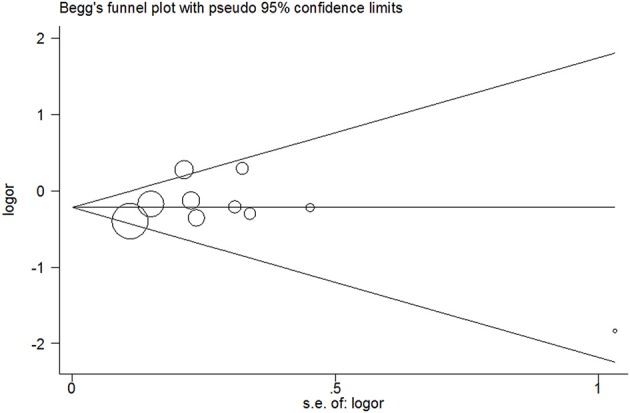
**Funnel plot for black tea consumption and risk of bladder cancer**.

**Figure 11:**
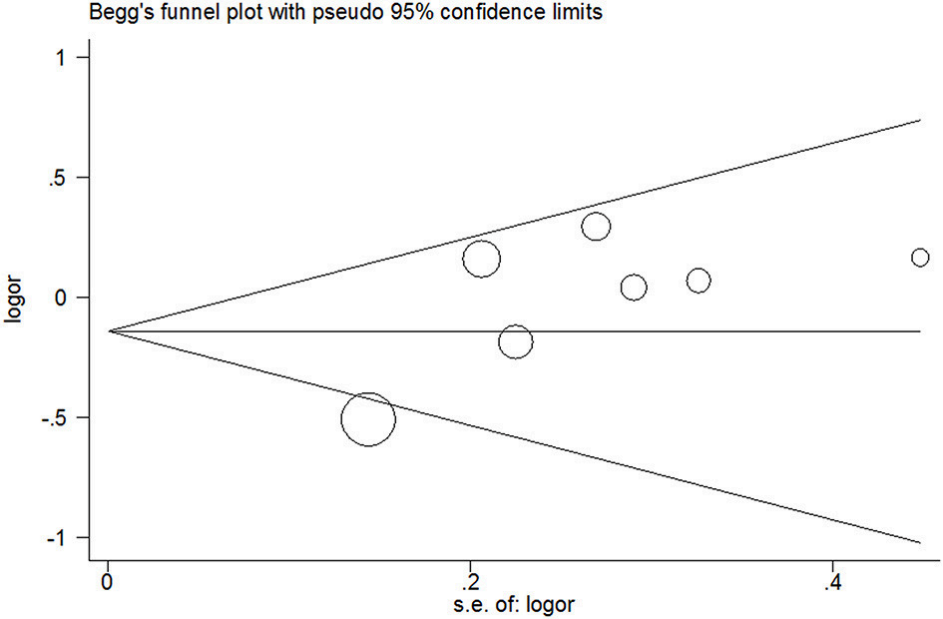
**Funnel plot for green tea consumption and risk of bladder cancer**.

No evidence of publication bias was detected in the tea (*P* = 0.43 for Egger's test; Figure 9), black tea (*P* = 0.76 for Egger's test; Figure 10), and green tea (*P* = 0.06 for Egger's test; Figure 11) consumption.

## Discussion

In present meta-analysis of seven prospective cohort studies that including 443 076 participants and 25 case-control studies that included 15 643 cases and 30 795 controls, we found that high level of tea intake was not significantly associated with bladder cancer risk. Specific analyses for black tea, green tea, and mate yielded similar results except for oolong tea. Dose-response analyses showed that there was no non-linearity or linearity association between tea intake and bladder cancer risk.

There are two concerns to the previously published meta-analysis. For instance, dose-response relationship were left unaddressed and only reported data for the highest vs. lowest comparison in two studies (Zeegers et al., 2001b; Qin et al., 2012), and type of tea was not considered in one study (Zeegers et al., 2001b). Accordingly, we performed this study with refined methods, and we obtained results consistent with the previous ones. Tea is a mixture of a large number of bioactive compounds. Certain laboratory studies in multiple animal models have suggested that green tea extract or polyphenols could inhibit the activity of tumor at different organ site (Yang et al., 2009; Yang and Wang, 2011). Therefore, it is not clear from these observational studies whether the tea extractor or polyphenols are beneficial and randomized controlled trials or controlled feeding studies would help examine this problem.

Several limitations should be taken into consideration for our study. First, limitations of observational studies contain the problem of residual confounding that may also extend to meta-analysis of observational studies (Threapleton et al., 2013). The quality and usefulness of any meta-analytic study are dependent on the quality and comparability of information from the individual studies (Hennekens and Demets, 2009; Zhou et al., 2011). Second, our meta-analysis included studies performed in different countries since the 1970s, and some studies had certain weakness in study design that were without stratification of type of tea, and some studies did not adjust confounders. Most of the studies included in this meta-analysis adjusted for important confounding factors such as age, sex, and smoking in their analyses, but not all studies adjusted for other potentially important variables such as occupation or other dietary factors (e.g., alcohol, coffee, vegetables, and fruits). However, the meta-regression and subgroup analyses based on those factors did not observe any difference. Additionally, bladder cancer is a complicated and heterogeneous disease, which is noted for marked global variations in incidence and etiology. Therefore, the results of the present meta-analysis should be considered with certain caution because of potential confounding. Third, measurement error in dietary assessment is an inherent problem when evaluating relationships between diet and diseases (Threapleton et al., 2013). The potential for exposure misclassification of tea consumption was also a limitation. The interval between lowest and highest categories was different among included studies. Moreover, the unit of tea consumption was also much different among identified studies. The aforementioned two factors may contribute to the heterogeneity among studies in the aggregated analysis. Indeed, we detected moderate to high heterogeneity among included studies. Fourth, as we all know, green tea is popular in East Asians such as Chinese and Japanese population, and the sample size of Asians in this meta-analysis was relatively small. Therefore, the selection bias was inevitable and the relationship among different countries still remained unclear. Lastly, we did not search for unpublished studies; therefore, even though no publication bias was detected through funnel plot and Egger's regression method, the publication bias might be inevitable. Lastly, the results of this meta-analysis showed that oolong tea consumption was associated with increased risk of bladder cancer. However, this finding was found only derived from one study (Lu et al., 1999). This intriguing finding wants further studies to investigate.

A major strength of this meta-analysis was the inclusion of observational studies from two main online database searches, identifying published studies from over two decades. The quality of the present meta-analysis was strengthened by assessing the quality of included studies and exploring dose-response relation not only reporting the comparisons of highest and lowest tea consumption. Different type of tea may have different influence. Therefore, we have evaluated the relationship between different type of tea and bladder cancer risk, which was an additional strength. Despite certain between-study heterogeneity was examined, the findings were generally consistent and the most of estimations were less than one. Therefore, drinking tea appears to be safe at habitual use.

In conclusion, this meta-analysis, consisting of 25 case-control studies and 7 cohort studies, had indicated that there was no significant association between tea consumption and the risk of bladder cancer. However, certain caution is need in interpreting the results from the present meta-analysis because of potential confounders and misclassification of tea consumption. Further studies with high quality and well-designed large scale are needed to provide more precise evidence.

## Author contributions

XW, HW, and XZ conceived the study. HW and JK searched the databases and extracted the data. HW, SL, and TL analyzed the data. HW and XZ wrote the draft of the paper. XW reviewed the manuscript. All the authors approved the final manuscript.

### Conflict of interest statement

The authors declare that the research was conducted in the absence of any commercial or financial relationships that could be construed as a potential conflict of interest. The reviewer Q.D. and handling Editor declared their shared affiliation, and the handling Editor states that the process nevertheless met the standards of a fair and objective review.
